# Perspectives of Persons With Disabilities Toward Home Adaptations and Assistive Products in Rural Northern Thailand: Comparative Study

**DOI:** 10.2196/79040

**Published:** 2025-10-23

**Authors:** Anuchart Kaunnil, Peeraya Munkhetvit, Hataichanok Apikomonkon, Jiranan Griffiths

**Affiliations:** 1 Department of Occupational Therapy, Faculty of Associated Medical Sciences, Chiang Mai University, 110 Intawaroros Rd., Sripoom subdistrict, Mueang District, Chiang Mai, 50200, Thailand, 66 53939259

**Keywords:** persons with disabilities, home adaptations, assistive products, rural Thailand, occupational therapy

## Abstract

**Background:**

Persons with disabilities in rural northern Thailand face significant challenges in accessing appropriate support, particularly in Chiang Mai’s rural community, where limited infrastructure and socioeconomic barriers hinder their independence. Home adaptations (HAs) and assistive products (APs) play a crucial role in enhancing the safety and quality of life; however, perspectives on their use remain underexplored.

**Objective:**

This study aimed to compare the views of persons with disabilities with and without experience of using HAs and APs, focusing on their perceptions of the environmental conditions, safety, benefits, and barriers.

**Methods:**

A comparative cross-sectional survey was conducted among persons with disabilities living in rural communities of the Doilor Subdistrict, Chiang Mai. Data were analyzed using descriptive statistics and the Mann-Whitney *U* test.

**Results:**

In 96 participants, 84 completed the questionnaire (48 without and 36 with experience using HAs and APs). Most individuals without HAs and APs were dissatisfied with their living conditions (36/48, 75%) and felt unsafe at home (33/48, 69%), whereas experienced users reported higher satisfaction (21/36, 58%) and safety confidence (25/36, 69%). While those without HAs and APs believed these adaptations could enhance their engagement at home, 75% (27/36) of experienced users reported that they had improved their participation skills. Significant differences were found between groups in perspectives on home conditions, living environment, and APs (*P*<.001), confidence in safety (*P*<.001), and skills for home engagement (*P*<.03). Both groups recognized the benefits of HAs and APs, with 94% (45/48) and 91% (33/36; without and with experience, respectively) agreeing that they improve daily functioning, and 95% (46/48) and 92% (33/36) acknowledging their role in reducing accident risks. HAs and APs significantly enhanced social participation (*P*<.04), with 41% (15/36) of experienced users strongly agreeing, compared with 19% (9/48) of those without experience. Financial constraints were the main barrier to HAs and APs adoption for over 90% of both groups. Service access challenges were more common among experienced users (32/36, 89%) than those without experience (36/48, 75%), with no significant differences in difficulties accessing services for home modifications and assistive devices.

**Conclusions:**

The study indicates that persons with disabilities face various problems. Both with and without experience of HAs and APs need to enhance their home modification and support to bridge the gaps in accessibility and practical solutions to improve the quality of life for persons with disabilities in rural communities. This study recommends that policymakers should focus on increasing funding, improving service delivery, and enhancing awareness programs to support individuals in need. Future research should explore the long-term impacts of HAs on quality of life and independence.

## Introduction

Over 1.3 billion people globally have some form of disability; these people represent about 16% of the global population [1]. The rise in noncommunicable diseases and longer life expectancy are contributing to a growing number of people with disabilities. This group is diverse, with their life experience and health needs shaped by various factors like sex, age, gender identity, sexual orientation, religion, race, ethnicity, and economic status. Furthermore, there were 190 million persons with disabilities aged 15 years and older who experienced obstacles in daily life and needed medical and social services [2]. Significantly, the majority of persons with disabilities, approximately 80%, reside in low- and middle-income countries, where they encounter substantial barriers to social participation and inclusion [3]. According to the countries in South America, housing modifications were established as sustainable development for persons with disabilities [4].

In Thailand, the National Statistical Office [5] conducts disability surveys every 5 years, with the most recent survey carried out between October and December 2022 to collect comprehensive data on persons with disabilities. It is estimated that 2.2 million persons with disabilities, representing 3.33% of the Thai population, comprise 51.4% (1,141,167/2,220,009) males and 48.6% (1,078,842/2,220,009) females. The most common types of disabilities are mobility disability (1,149,437/2,220,009, 51.78%), followed by hearing impairment (428,397/2,220,009, 19.3%) and visual impairment (171,083/2,220,009, 7.71%). Regarding labor force participation, 48.72% (395,252/811,350) of persons with disabilities are unemployed, 26.84% (217,737/811,350) are unable to work due to severe disabilities, 24.09% (195,434/811,350) are employed, and some prefer not to disclose their occupation. Most persons with disabilities are employed in the agriculture sector (96,153/195,434, 49.2%), followed by the service and trade sectors (75,828/195,434, 38.8%) and the production sector (23,452/195,434, 12%) [5]. As a result, these persons with disabilities face barriers to employment, leading to a diminished quality of life. According to the World Health Organization (WHO) [6], quality of life is influenced by the interaction between an individual, social participation, and their environment. Five key environmental factors contributing to this interaction include (1) products and technology, (2) the built environment, (3) social relationships and support, (4) societal attitudes, and (5) services, systems, and policies. Within the various factors influencing daily living, environment and products are integral components within the broader domain of assistive products (APs) and technology. This is a field that plays a crucial role in enhancing quality of life by promoting independence, enabling active participation, and supporting overall well-being among persons with disabilities, older adults, and other populations in need.

Home adaptations (HAs) refer to permanent structural modifications made to enhance accessibility and suitability for individuals with disabilities [7]. This intervention aims to prevent further deterioration of bodily functions, enhance functional capacity, and promote engagement in social and community services. The primary goal of HA is to eliminate environmental barriers, enabling individuals to perform daily activities with greater safety and efficiency. This approach aligns with environmental theories emphasizing the relationship between the individual, their environment, and functional performance. Research conducted in Sweden demonstrated that HAs significantly improved individuals’ abilities to perform daily living activities and enhanced their sense of safety [8]. In addition, a separate study found that environmental assessments and modifications could effectively reduce care needs among older people and persons with disabilities [9].

APs encompass a wide range of external devices such as prosthetic limbs, wheelchairs, hearing aids, pill organizers, and accessible information and communication technology that are designed to support persons with disabilities by enhancing their functional independence and ability to participate in daily life activities [10]. However, just 10% of people worldwide have access to APs, making the proportion of persons with disabilities who do so extremely low. To address this, the WHO launched the Global Cooperation on Assistive Technology initiative and the Priority Assistive Products List to improve access through better infrastructure, policies, service delivery, and trained personnel [11]. Furthermore, Tebbutt et al [12] found that without universal access to APs, the Sustainable Development Goals cannot be achieved equitably, reinforcing their role as a cornerstone of inclusive and sustainable development.

In previous studies, Ding et al [13] examined practitioners’ perspectives on the growing affordability (budget-friendly) and practical importance of HAs, smart technology, and assistive devices. Their findings indicate that these interventions are vital in assisting persons with disabilities by improving environmental management and promoting greater independence [13]. Giesbrecht et al [14] examined the mobility device needs of Canadians with disabilities (n=45,442) using follow-up data from the National Household Survey. They found that 10% of wheeled mobility device users had unmet needs, with users experiencing higher unmet needs than nonusers. In addition, users required more home modifications and assistance compared with nonusers. According to Labbé et al [15], the research team explored how families with disabilities perceive their homes, using the psychoenvironmental potential model to assess home characteristics influencing well-being. Through interviews with 31 individuals with spinal cord injuries, they found that home plays a crucial role in meeting psychological and social needs. Their findings highlight the significant impact of home on well-being and suggest that disability shapes experiences differently for individuals and families.

Struckmeyer et al [16] examined home modifications concerning accessibility challenges and aesthetic preferences. Through focus groups involving 16 participants (8 consumers and 8 professionals), the study identified barriers to accessibility, including inadequate contrast, inappropriate fixture heights, door designs, and flooring. In addition, findings indicated that consumers prioritize attractiveness more than professionals. The study suggests that repurposing existing spaces can effectively address affordability while accommodating consumer preferences, consistent with Ainsworth et al [17], who conducted a study to examine the experiences of older adults and persons with disabilities regarding home modifications and their perceived value of outcomes. Using an interpretive description approach, interviews were conducted with 20 participants aged 24-93 years. The findings revealed two primary themes: (1) experience before and during home modifications and (2) experience after modifications. The study highlighted that valued outcomes included enhanced health, safety, and considerations for future planning.

In South Korea, Jo and Kim [18] conducted a pilot randomized controlled trial comparing the effects of HAs and APs with home exercise among 20 persons with disabilities. The findings indicated that the use of HAs and APs significantly increased activities of daily living participation time in the experimental group (*P*<.05). Furthermore, the experimental group demonstrated enhanced competence in occupational performance, while the control group exhibited improved occupational performance values (*P*<.05). In addition, activity limitations significantly decreased in the control group (*P*<.05), whereas no significant reduction was observed in the experimental group.

In Thailand, Sukkay [19] conducted a study on multidisciplinary approaches to HA design for persons with disabilities, using the International Classification of Functioning, Disability and Health framework in conjunction with postoccupancy evaluations focused on mobility. The study revealed that the majority of residencies of persons with disabilities lacked standardized criteria for HAs. Notably, inappropriate room dimensions and unsuitable furniture significantly impeded accessibility. The study emphasized that essential spaces, such as toilets and bedrooms, were particularly difficult for persons with disabilities to access. Building upon this work, Sukkay and Upala [20] developed a set of housing and spatial design guidelines tailored for persons with disabilities. Their methodology involved a comparative assessment of guideline approaches—one based on comparative studies, and the other on participatory processes. Data were collected via questionnaires distributed to 30 government officials and 30 individuals with mobility impairments. The findings indicated significant differences in the evaluation of design categories between the 2 groups. Furthermore, the participatory-based guideline approach demonstrated greater efficacy in addressing user needs than the comparative study-based method.

While Tongsiri et al [21] examined the effectiveness of a home environment modification program aimed at improving the quality of life for Thai persons with disabilities, their intervention incorporated training sessions focused on universal design principles, followed by direct modifications in 43 homes. The results underscored the importance of collaboration with district hospitals and local health care teams to optimize the functional capacities of individuals with disabilities. More recently, Selanon and Chuangchai [22] explored interior residential design preferences and needs among individuals with physical disabilities. Employing a large-scale questionnaire survey comprising 384 participants with 8 types of disabilities, their study found that physical abilities varied significantly by age, gender, and place of residence. Importantly, the data revealed a positive correlation between in-home mobility and various outcome measures such as out-of-home mobility, physical capabilities, independence, and perceived health status (*P*<.05). These findings highlight the critical need for residential interior design that accommodates physical variability through strategic planning of layout, furniture, flooring, and lighting to enhance overall mobility.

Hence, Thai persons with disabilities continue to face significant barriers in accessing HAs and APs, despite national efforts to promote inclusive living environments. Previous studies had identified substantial gaps in the availability and accessibility of HAs and APs support services, underscoring the pressing need for strengthened policies to ensure equitable access to health care and social support for persons with disabilities [23,24]. Furthermore, studies have pointed to low rates of disability registration and limited uptake of disability-related welfare benefits, suggesting systemic challenges in reaching persons with disabilities and their families through current service delivery models [25].

In terms of HAs and APs approach, occupational therapists play a critical role in assessing an individual’s capacity to perform daily living activities through comprehensive activity analysis. This process involves evaluating and recommending appropriate HAs and assistive devices tailored to the client’s needs, skills, and abilities. Occupational therapy interventions frequently include structural modifications such as installing grab bars, ramps, walking aids, bath transfer cushions, and pressure-relieving cushions. These modifications aim to create a more accessible and safer environment, promote independent self-care, enhance access to household items and furniture, and ultimately improve overall quality of life [26]. By integrating knowledge of daily living activities, environmental modifications, and assistive technology, occupational therapists strive to align interventions with the individual’s lifestyle, personal factors, environment, and broader contextual influences.

This study focused on the target area at Doilor Subdistrict, situated in Chiang Mai Province in northern Thailand. This rural area comprises 26 villages with a total population of 12,227 individuals, including 5982 males and 6245 females [27]. Most residents (8546/12,227, approximately 70%) are engaged in agricultural work, producing crops such as longan, rice, cantaloupe, tomato, and pumpkin, with longan being the primary export commodity. As of 2024, a total of 235 persons with disabilities were officially registered in Doilor community [27]. Access to appropriate HAs and APs plays a vital role in enhancing the health, autonomy, and overall quality of life of persons with disabilities, particularly in rural and remote areas. By exploring the perspectives of persons with disabilities in this rural northern Thai community, this study aims to compare the views of persons with disabilities with and without experience using HAs and APs, focusing on their perceptions of environmental conditions, safety, benefits, and barriers. This study could contribute to the development of more responsive, community-centered strategies that bridge the gap between policy and practice.

## Methods

### Overview

A cross-sectional study was designed using a questionnaire to examine the perspectives of people with disabilities on HAs and APs. A structured questionnaire was developed comprising four main sections: (1) participant background—collected demographic and general information about the respondents; (2) perceived benefits of HAs and APs—assessed participants’ views on the advantages and usefulness of HAs and APs in daily living; (3) accessibility and usability—examined the availability, ease of access, and usability of HAs and APs in participants’ homes and communities; and (4) challenges and barriers—identified obstacles and limitations faced by persons with disabilities in adopting or using HAs and APs. Before its deployment, the questionnaire underwent validation using the item-objective congruence method to evaluate content relevance and clarity. Each item was rated on a scale from –1 to +1, where +1 indicated clear relevance (congruent), 0 indicated uncertainty (questionable), and –1 indicated irrelevance (incongruent). Items scoring 0.5 or higher were retained. Those with lower scores were either revised based on expert feedback or removed if no feedback was available. Content validation was conducted by 3 licensed occupational therapists, each with over a decade of experience working with persons with disabilities in rural Thai communities. Following expert review and revision, the final version of the questionnaire achieved an item-objective congruence index score of 0.83, indicating strong content validity. Participants responded to survey items using a five-point Likert scale: (1) strongly disagree, (2) disagree, (3) neutral, (4) agree, and (5) strongly agree.

The required sample size was calculated with Cochran’s sample size formula for proportion [28]. The total persons with disabilities population in Doilor subdistrict, Chiang Mai Province, was obtained from the database (n=235). The Cochran sample size formula was modified for a smaller (finite) population, resulting in a sample size of 91. After adding 5% for the nonresponse rate, the final sample size was calculated to be 96 persons with disabilities.

The study used a structured approach to data collection. Between October and November 2022, the research team visited the Doilor Community Rehabilitation Center to administer questionnaires to persons with disabilities who met the inclusion criteria. Questionnaires and informed consent forms were provided for participants at home. All participants reviewed and signed the consent forms before completing the questionnaires. Additional information was collected regarding the participants’ home settings and surrounding environments. To ensure the quality and reliability of the data, each step of the research process was carefully documented.

The study analyzed survey data using descriptive statistics and employed the Mann-Whitney *U* test to compare median responses on Likert scale items between 2 independent groups. Data analysis was conducted using SPSS version 25.0 for Windows (SPSS Inc). Given the nature of the data, nonparametric tests were used to compare participants with and without experience using home HAs and APs. The Mann-Whitney *U* test assessed group differences, with statistical significance set at *P*<.05 (1-tailed).

### Ethical Considerations

The study was approved by the Ethics Committee of the Faculty of Associated Medical Sciences at Chiang Mai University (reference AMSEC-65EX-030). Before participation, written informed consent was obtained from the legally authorized representatives of all participants. The data safeguarded participants’ privacy, maintained the confidentiality of all information collected, and ensured fair compensation for their involvement.

## Results

A questionnaire survey was conducted with 96 participants from the Doilor community. A total of 84 individuals participated and completed the questionnaire, including 37 (44%) men and 47 (56%) women. Among them, 35 (42%) participants were older than 60 years old, 21 (25%) were aged 51‐60 years, and 18 (21%) were aged 41‐50 years. Regarding marital status, 53% (43/84) were married, 42% (34/84) were widowed, and 2.5% (2/84) were separated. Most participants (65/84, 77%) lived alone, while 23% (19/84) lived with family members; 88% (74/84) resided on their own land. In terms of education, 59% (49/84) had completed primary school, while 35% (29/84) had no formal education. More than half (44/84, 52%) were still employed. Most participants (31/84, 70%) were employed by others and received wages or salaries: 21% (9/84) engaged in farming on their own land or family-owned farms, and 9% (4/84) ran small businesses, such as local shops or self-employed services. Notably, 92% (77/84) reported a monthly income below 5000 Baht (approximately US $149), as detailed in Table 1.

**Table 1.** Demographic data of the survey participants (n=84).

Categories	Participants, n (%)
Gender	
Women	47 (56)
Men	37 (44)
Total	84 (100)
Age (y)	
18‐30	4 (5)
31‐40	6 (7)
41-50	18 (21)
51-60	21 (25)
>60	35 (42)
Marital status	
Married	43 (53)
Widow or widower	34 (42)
Divorced	2 (2.5)
Separated	2 (2.5)
Living arrangement	
Family	19 (23)
Alone	65 (77)
Residency status	
Owner	74 (88)
Resident with family	10 (12)
Rent	—^a^
State properties	—
Educational level	
Uneducated	29 (35)
Primary school	49 (59)
Junior high school	2 (2)
Senior high school	3 (4)
University degree	—
Work status	
Work	44 (52)
Retired or unemployed	40 (48)
Profession status (n=44)	
Agriculture (work on their own land or family-owned farms)	9 (21)
Business (local shops or self-employed services)	4 (9)
Employee (are employed by others and receive wages or salaries)	31 (70)
Income (month)	
<5000 Baht (US $149)	77 (92)
5000‐10,000 Baht (US $149‐298)	7 (8)
10,001‐15,000 Baht (US $298‐447)	—
>15,000 Baht (US $447)	—

a Not applicable.

The survey results revealed that 57% (48/84) of participants without previous experience using HAs and APs (nonusers), while 39% (36/84) had experience using HAs and APs (users). Among those who had access to these HAs and APs, the majority (21/84, 58%) received them from family members, while 39% (14/84) were supported by government assistance.

Multimedia Appendix 1 presents the distribution of daily living activities supported by HAs and APs. Toileting emerged as the most frequently supported activity, reported by 39.2% (33/84) of participants. This was followed by bathing and showering (22/84, 25.7%), and indoor and outdoor mobility devices, including wheelchair use (9/84, 10.8%). A smaller proportion of participants reported support for activities such as health management, personal hygiene and grooming, and dressing, with each category accounting for only 1.4% (1/84) of responses, as presented in detail in Multimedia Appendix 2.

Table 2, participants without previous experience using HAs and APs, largely expressed dissatisfaction with their home environments. Specifically, 35% (17/48) strongly disagreed and 40% (19/48) disagreed with statements indicating satisfaction with their home conditions and AP availability. Furthermore, 27% (13/48) strongly disagreed and 42% (20/48) disagreed that their homes were safe for performing daily activities. Conversely, participants who had previous experience with HAs and APs demonstrated significantly more positive attitudes. Specifically, 36% (13/36) reported agreement and 22% (8/36) reported strong agreement regarding satisfaction with their home environment. In addition, 69% (33/48) reported feeling safe while using HAs and APs. Among participants without experience in HAs and APs, 69% (33/48) agreed and 21% (10/48) strongly agreed that they felt confident in their abilities, while participants with experience in HAs and APs presented 58% (21/36) agreeing and 32% (12/36) strongly agreeing.

**Table 2.** Participants’ perspectives on their current home, living environment, and use of various assistive products (n=84), including participants without experience home adaptations and assistive products (n=48) and participants with experience home adaptations and assistive products (n=36).

Perspective and subgroup	Strongly disagree, n (%)	Disagree, n (%)	Neutral, n (%)	Agree, n (%)	Strongly agree, n (%)
Do you think that now your home, living environment, and various APs^a^ are already in good condition?					
Without experience HA^b^ and AP	17 (35)	19 (40)	1 (2)	6 (13)	5 (10)
With experience HA and AP	4 (11)	4 (11)	7 (20)	13 (36)	8 (22)
Are you confident that your home, living environment, and APs are safe for activities and use?					
Without experience HA and AP	13 (27)	20 (42)	2 (4)	9 (19)	4 (8)
With experience HA and AP	2 (6)	3 (8)	6 (17)	13 (36)	12 (33)
Do you currently feel like you have the skills you need to use your home, living environment, and APs?					
Never used HA and AP	11 (23)	4 (8)	5 (11)	24 (50)	4 (8)
Having experience with HA and AP	2 (6)	3 (8)	4 (11)	20 (56)	7 (19)
Do you think you could improve your activity skills if your living environment were adapted, and APs were provided for you?					
Never used HA and AP	3 (6)	2 (4)	0 (0)	33 (69)	10 (21)
Having experience with HA and AP	0 (0)	2 (5)	2 (5)	21 (58)	12 (32)

a AP: assistive product.

b HA: home adaptation.

The Mann-Whitney *U* test results in Table 3 reveal significant differences in participants’ perspectives between those without and with experience using HAs and APs. Participants with experience using HAs and APs reported more positive views regarding the condition of their home and assistive environment (*P*≤.001), greater confidence in the safety of their surroundings (*P*<.001), and higher self-rated skills in using these supports (*P*=.03). However, no significant difference was found between the groups in their perceived potential for activity improvement through APs and environmental changes (*P*=.41).

**Table 3.** Mann-Whitney *U* test results comparing participants without and with experience on perspectives of their current home, living environment, and use of various assistive products.

Topics	*U* value	*P* value
Condition of home, living environment, and assistive products	4590	<.001^a^
Confidence in the safety of home and assistive products	376.5	<.001^a^
Skills to use home, living environment, and assistive products	646.0	.03^a^
Potential activity improvement with environmental adaptations and assistive products	785.5	.41

a Significant *P* value <.05, the Mann-Whitney *U* test was applied.

As illustrated in Table 4, both participant groups without and with experience using HAs and APs recognized their benefits. A majority agreed that HAs and APs could help restore daily functioning (27/48, 56% agreed and 18/48, 38% strongly agreed among nonusers; 17/36, 47% agreed and 16/36, 44% strongly agreed among users) and improve efficiency and quality of life (31/48, 65% agreed and 15/48, 31% strongly agreed; 21/36, 58% agreed and 10/36, 28% strongly agreed, respectively). Comparable trends were observed regarding time savings (30/48, 63% agreed and 15/48, 31% strongly agreed among nonusers; 19/36, 53% agreed and 12/36, 33% strongly agreed among users) and reduction of accident risks (31/48, 64% agreed and 15/48, 31% strongly agreed among nonusers; 20/36, 56% agreed and 13/36, 36% strongly agreed among users). With respect to social participation, respondents believed that HAs and APs supported engagement in family and social activities (36/48, 75% agreed and 9/48, 19% strongly agreed among nonusers; 19/36, 53% agreed and 15/36, 41% strongly agreed among users). Notably, persons with disabilities without experience using HAs and APs consistently reported higher levels of agreement across all categories.

**Table 4.** Participants’ perspectives on the benefits of modifying the home environment and using assistive products (n=84), including participants without experience home adaptations and assistive products (n=48) and participants with experience home adaptations and assistive products (n=36).

Perspective and subgroup	Strongly disagree, n (%)	Disagree, n (%)	Neutral, n (%)	Agree, n (%)	Strongly agree, n (%)
Do you think that modifying your home environment and using APs^a^ can help you regain the ability to perform daily activities?					
Without experience HA^b^ and AP	0 (0)	2 (4)	1 (2)	27 (56)	18 (38)
With experience HA and AP	0 (0)	1 (3)	2 (6)	17 (47)	16 (44)
Do you believe that modifying your home or living environment, and using APs can help you improve your efficiency and quality of life?					
Without experience HA and AP	0 (0)	2 (4)	0 (0)	31 (65)	15 (31)
With experience HA and AP	0 (0)	1 (3)	4 (11)	21 (58)	10 (28)
Would you be able to save time on your daily activities if your home environment was modified and you had access to various APs?					
Without experience HA and AP	0 (0)	1 (2)	2 (4)	30 (63)	15 (31)
With experience HA and AP	0 (0)	0 (0)	5 (14)	19 (53)	12 (33)
Do you believe that modifying your home, living environment, and APs can help you reduce the risk of accidents or injuries?					
Without experience HA and AP	0 (0)	1 (2)	1 (2)	31 (64)	15 (31)
With experience HA and AP	0 (0)	2 (5)	1 (3)	20 (56)	13 (36)
Do you think that modifying your home, living environment, and using APs can help you participate more in family activities and activities with others?					
Without experience HA and AP	0 (0)	1 (2)	2 (4)	36 (75)	9 (19)
With experience HA and AP	0 (0)	1 (3)	0 (0)	19 (53)	15 (41)

a AP: assistive product.

b HA: home adaptation.

According to the Mann-Whitney *U* test results in Table 5, participants with experience in using HAs and APs significantly differed from those without experience only in their views on increasing participation in family and social activities (*P*=.04). For other perceived benefits such as regaining the ability to perform daily activities, improving efficiency and quality of life, saving time, and reducing the risk of accidents, no significant differences were found between the 2 groups (*P*>.05).

**Table 5.** Mann-Whitney *U* test results comparing participants without and with experience on the benefits of modifying the home environment and using assistive products.

Topics	*U* value	*P* value
Regaining ability to perform daily activities	817.5	.64
Improving efficiency and quality of life	946.5	.39
Saving time on daily activities	890.0	.79
Reducing the risk of accidents and injuries	849.0	.88
Increasing participation in family and social activities	674.0	.04^a^

a *Significant *P *value <.05, Mann-Whitney *U* test was applied.

Multimedia Appendix 2 outlines the perceived barriers to participants without and with experience using HAs and APs. Financial constraints emerged as the most significant obstacle, with over 90% (47/48) of respondents identifying cost as a major concern (11/48, 23% agreed and 36/48, 75% strongly agreed among nonusers; 8/36, 22% agreed and 26/36, 72% strongly agreed among users). A substantial proportion also cited a lack of information regarding HAs and APs (39/48, 82% agreed and 5/48, 10% strongly agreed for nonusers; 26/36, 72% agreed and 5/36, 14% strongly agreed for users). Difficulties in accessing HAs and APs services were reported by 80% of both groups (34/48, 71% agreed and 6/48, 13% strongly agreed among nonusers; 28/36, 78% agreed and 4/36, 11% strongly agreed among users). Concerns about maintenance were also prevalent, although more pronounced among users (33/48, 68% agreed and 10/48, 20% strongly agreed for nonusers; 16/36, 45% agreed and 13/36, 36% strongly agreed for users). While the Mann-Whitney *U* test found no significant differences between participants without and with experience regarding perceived barriers to HAs and APs. Both groups reported similar concerns across all topics, including cost, information, service access, and maintenance, with only service access nearing significance (*P*=.05).

## Discussion

### Principal Findings

This study explored the experiences of persons with disabilities in a rural Thai community (Doilor subdistrict) regarding HAs and APs, which are individualized for each participant. The findings contribute valuable insights to the understanding of HAs and APs for persons with disabilities. In this study, most participants without HAs and APs were dissatisfied with their living conditions (36/48, 75%) and felt unsafe at home (33/48, 69%), while those with HAs and APs experience reported greater satisfaction (21/36, 58%) and safety (25/36, 69%). According to several studies, home modifications and APs can enhance the satisfaction of persons with disabilities in daily life, improve postmodification safety, and positively impact their mental well-being by reducing feelings of insecurity at home [29,30]. Importantly, this study found that participants with experience using HAs and APs had shown more positive perceptions of their home environment (*P*<.001), greater confidence in its safety (*P*<.001), and stronger self-rated usage skills (*P*=.03). However, no significant differences emerged between the groups in terms of their perceived potential for improving activity skills, such as functional mobility (eg, moving safely within the home), self-care tasks (eg, bathing, dressing, and feeding), and household activities (eg, cooking and cleaning); through APs and HAs (*P*=.41). This finding is noteworthy as it indicates that both groups equally acknowledged the value of these interventions in promoting engagement in daily life. The convergence of views suggests a shared understanding among persons with disabilities of the essential role that HAs and APs play in fostering independence, safety, and participation in meaningful activities. Furthermore, the agreement across groups regarding the potential for increased participation in family and social activities underscores the broader social implications of such interventions. Consistent with previous studies [31,32], these findings reinforce the importance of integrating home modifications and assistive technologies into rehabilitation and community-based support strategies. Regardless of differences in demographic or functional characteristics, the need for accessible and supportive home environments remains a priority for improving the quality of life of persons with disabilities in rural contexts.

In this study, persons with disabilities also believed that using HAs and APs contributed to the restoration of daily functioning (45/48, 94% among nonusers and 33/36, 91% among users) and enhanced efficiency and quality of life (46/48, 96% among nonusers and 31/36, 86% among users). According to Moon et al [30], the implementation of the Korean smart home modification program and devices assisted individuals with physical disabilities in maintaining occupational performance and improving their engagement in daily activities, ultimately promoting a higher quality of life. Furthermore, this study found that persons with disabilities perceived that using HAs and APs could support time (45/48, 94% among nonusers and 31/36, 86% among users) and reduce the risk of accidents or injuries (46/48, 95% among nonusers and 33/36, 92% among users). Consistent with Petersson et al [8], this study explored how home modifications influence the ability of persons with disabilities to carry out daily tasks. The findings demonstrated that home modifications not only alleviate difficulties in performing everyday activities but also result in measurable time savings within 6 months. Similarly, Jo and Kim [18] implemented occupation-based home modifications for persons with disabilities, demonstrating improvements in time efficiency, participation in activities of daily living, and occupational performance. Furthermore, the innovative safe at home provided various in-home accessibility enhancements such as grab bars, safety railings, stair lifts, and bathtub cutouts for older adults and individuals with disabilities. This study revealed high program satisfaction (190/241, 79%), an absence of falls, and a notable reduction in fear of falling following the use of APs and environmental modifications [33]. In contrast, our study observed no significant differences between groups (without and with experience using HA and AP) regarding perceived improvement in daily function, efficiency, quality of life, timesaving, or accident and injury prevention.

In our study, participants with previous experience using HA and AP reported that such modifications, along with improvements in the living environment, significantly enhanced their engagement in family and general activities (15/36, 41% strongly agreed while 19/36, 53% agreed. In contrast, among participants without experience with HA and AP, only 19% (9/48) strongly agreed and 75% (36/48) agreed with this sentiment. Goddard et al [29] observed that individuals with mobility impairments perceived home modifications as beneficial in reducing physical exertion while enhancing social interaction and independence. Similarly, Greiman et al [34] emphasized that improved home usability enables access to appropriate housing, thereby promoting community participation among individuals with disabilities. Their findings further suggested that enhanced home usability facilitates participation by improving health, autonomy, social interaction, organization, and safety. This is confirmed that our study of both groups of participants presented significant differences in relation to increasing participation in family and social activities (*P*=.04).

The key barrier reported by participants identified financial constraints as the primary obstacle to modifying homes and acquiring affordable APs reported by 98% (47/48) of nonusers and 94% (34/36) of users. Gusheh et al [35] noted that most persons with disabilities were compelled to self-finance modifications, which were frequently unaffordable, especially for individuals with constrained financial resources. Layton et al [36] similarly assert financial limitations significantly impede the availability and implementation of HAs and APs for persons with disabilities. Existing funding structures often fall short in accommodating the necessary adaptations, thereby fostering inequities in access to essential resources. Consistent with Puli et al [37], financial exclusion continued to impact persons with disabilities in low- to middle-income countries. Although the demand for affordable and accessible housing is rising, current financial mechanisms remain insufficient to meet these needs. Many families face financial constraints that limit their ability to afford necessary HAs and APs. In addition, there is often a lack of availability and accessibility of these products in rural areas, which poses a significant barrier to improving the living conditions of persons with disabilities [26,32]. In the long term, the effectiveness of sustainable approaches to addressing this issue has been demonstrated in a study conducted in India [38]. Prajapati et al [38] highlight that integrating persons with disabilities into the economic structure could increase national gross domestic product by 3%‐7%, demonstrating their potential as active contributors rather than passive beneficiaries. Furthermore, industry collaboration, start-ups, and public-private partnerships could enhance access to affordable assistive technologies while also generating employment opportunities for persons with disabilities. Importantly, initiatives such as academia-industry collaboration, corporate social responsibility funding, and innovation parks have been shown to support sustainable models of device development and distribution. Thailand should learn from and adapt these strategies to effectively address the financial limitations of persons with disabilities.

The lack of mandatory accessibility standards in residential construction continues to reinforce the prevalence of inaccessible housing, highlighting the need for greater awareness about HAs and APs. Our study revealed that a substantial proportion of individuals face difficulties accessing HAs and APs services (40/48, 84% among nonusers and 32/36, 89% among users). In addition, a widespread lack of information was observed, especially among participants with no previous experience: 92% (44/48) reported insufficient knowledge, compared with 86% (31/36) among those with previous experience. According to the studies, evidence from both low- and middle-income countries indicates that persons with disabilities often lack awareness of available HAs and APs, as well as knowledge on how to access or use them. In Nigeria, for instance, Okonji and Ogwezzy [39] found that only 36% (152/423) of visually impaired adults were aware of assistive technologies, and just 17.4% (74/423) reported knowing how to use them. Similarly, a study by Senjam et al [40] in India revealed that among visually impaired patients, high levels of awareness were observed for only 2 out of 42 assistive devices, with more than 30 devices showing awareness levels below 33%. These findings underscore a critical gap in information that must be addressed by health care providers, rehabilitation professionals, and policymakers. Enhancing awareness and facilitating the use of APs and technologies should be a priority in service delivery.

Beyond statutory schemes, Thailand has an active system of nongovernmental organizations (NGOs) and foundations that raise awareness and enable practical access to HAs and APs in rural communities. The Mahasarakham University Universal Design Center’s Home for Life program trains multidisciplinary local teams (public health staff, civil servants, and built-environment actors) to identify hazards and plan universal-design home changes; the program has been scaled with Thai Health Promotion Foundation support and a train-the-trainer model (improving local capacity for sustainable adaptations) [41]. The Rajanagarindra Institute of Child Development Wheelchair Project sources and customizes wheelchairs and mobility aids that often require complementary home changes (eg, ramps and bathroom adaptations) supplies adapted mobility and daily living devices through community programs [42]. Community-based organizations such as Buddy HomeCare coordinate home-based support and link clients to product or adaptation options, frequently acting as catalysts for simple, affordable environmental modifications [43]. The initiatives led by these NGOs and foundations enhance awareness, facilitate referrals, and mobilize community resources, providing practical pathways to overcome the access barriers identified in the Thai sample.

Furthermore, persons with disabilities and their families frequently report a lack of clarity on how and where to obtain reliable information on home and APs. As highlighted by Jutai et al [44], individuals with low income and disabilities often face compounded barriers due to both limited access to information and insufficient resources for device acquisition. In many cases, the absence of centralized or accessible information emerges as a primary obstacle to the broader adoption of APs. This trend is evident across diverse settings; for example, in Bangladesh, Myanmar, and similar contexts, Pryor et al [45] report that many individuals remain unaware of available devices, services, or even the existence of disability support programs. This systemic lack of information presents a major challenge to equitable access and usage of home modification and device support. Although our study found no statistically significant differences between participants with and without previous experience regarding barriers to HAs and APs, the difficulty in accessing services approached significance (*P*=.05). This implied that some users with experience of HAs and APs may have slightly better knowledge or access to services, which could explain the small difference between the groups.

Maintaining home and environmental adaptations, as well as APs, poses a significant challenge for persons with disabilities and their families. In this study, 88% (43/48) of participants without experience using HAs and APs, and 81% (29/36) of those with experience reported difficulties in maintaining them. This highlights the difficulty of repairing or maintaining these devices at home, with users struggling to access appropriate repair services, ensure sustainability, and manage upkeep. Toro Hernandez [46] found that among 142 Indonesian wheelchair users, 34% (n=142) required more than one repair after receiving a new wheelchair, and 70% (n=142) did not receive complete repairs—underscoring a gap in maintenance training. Similarly, Eide et al [47] found that in low-income countries, only 7% of repairs were handled by government services, while 40% were managed by users or their families; another 40% of devices remained unrepaired due to financial constraints and other barriers. While Oldfrey et al [48] conducted a synthesis of findings across 4 distinct studies and contextual settings, their analysis revealed that, in low-resource environments, the scarcity of specialized repair services poses a significant barrier to the effective provision of assistive devices and technology. In such contexts, community-based repair practices often serve as the primary means of device maintenance. To improve the efficacy of assistive devices and technology delivery, formal systems should recognize, incorporate, and support these grassroots repair mechanisms. Nonetheless, a critical gap persists in the availability of data tracking AP outcomes over their lifecycle, particularly in areas such as follow-up, repair, and maintenance, which hampers further advancements. Strengthening community-based repair networks may also facilitate more accurate referrals to specialist services when complex repairs are needed.

The quality and appropriateness of HAs and APs can also be strengthened through 3 mutually reinforcing strategies. First, copartnerships among local small and medium-sized enterprises, universities, and clinical services should collaborate with the industrial sector to identify relevant APs related to the needs of persons with disabilities and users in the communities [49]. Integrating suppliers for prequalification, product standards, and service standards (eg, WHO guidance for priority APs and wheelchair provision) should be implemented to ensure the safety and maintenance of after-sale support in rural communities [50,51]. Second, product development which embraces ramps, low-cost transfer aids, context-appropriate wheelchairs, bathroom modifications, and toilet adaptations should be designed in alignment with the local context and combined with local materials including easy repair [52]. This development should be coordinated with social enterprises and public agencies [53]. Third, training programs should be implemented to develop local staff competencies that include (1) community workers for screening and follow-up; (2) technicians for fitting, customizing, and fixing and repairing; and (3) therapists for assessment, evaluation, prescription, fitting, and monitoring. These strategies could support persons with disabilities in the needs of HAs and APs based on the local contexts [54].

Even though subsidies could reduce immediate financial limitations of the families of persons with disabilities, they are not a long-term solution for ensuring equitable access to HAs and APs in the low- and middle-income countries [55,56]. Subsidy approaches by public agencies could fail due to the weak service capacity, supply chains, and follow-up and maintenance systems. Sustainability should be coordinated based on community-based rehabilitation, NGOs, and private partnerships. This would build local capacity through train-the-trainer models and strengthen the sustainable community mobilization [57]. In APs such as wheelchairs, the use of the WHO Wheelchair Service Training Package needs to be used in service step assessment, fitting, training, and follow-up. This subsidy and training program could enhance the ability of persons with disabilities to meet their outcomes and reduce the condition of dependence and abandonment [58]. The use of local materials in manufacturing and designs could reduce costs and increase supply chains [59]. Effective procurement, outcome-based contracts, and microcredit with transparency could allow the families of persons with disabilities to access the money with accountability [60]. Finally, integrating a competency-based workforce for persons with disabilities into employment will be a successful process that is supported by mentoring under therapists and stakeholders to ensure long-term usability. These approaches should be reinforced to provide service delivery and supply, quality, and local ownership to remove obstacles and promote independence for persons with disabilities.

In Northern Thailand, HAs and APs are vital for promoting mobility, independence, and the overall well-being of persons with disabilities in rural areas. Nevertheless, access to these essential supports is significantly hindered by geographic isolation, limited health care infrastructure, and socioeconomic constraints. Homes often have hazardous features, such as inappropriate door widths and high thresholds, which can impede mobility and independence for individuals with disabilities. These environmental barriers highlight the need for targeted home modifications [61]. Although global progress has been made in advancing disability-inclusive policies, rural regions in low- and middle-income countries often remain underserved due to persistent economic, infrastructure, and sociocultural barriers. While HAs and APs are instrumental in facilitating daily activities, a pronounced disparity between urban and rural access persists, with rural persons with disabilities facing greater obstacles in obtaining these resources. To effectively address this challenge in rural Northern Thailand, local municipalities, governmental and private agencies, and NGOs, including industrial sectors, should not only provide targeted subsidies but also strengthen the ability and capability of persons with disabilities to use HA and APs.

### Limitations

The primary limitation of this study lies in its recruitment from a single subdistrict (Doilor community), which restricts the generalizability of the findings to the broader population of persons with disabilities in rural Thailand. In addition, the reliance on self-reported data may have introduced social desirability bias, potentially leading to an overestimation of the perceived benefits of HAs and APs. The study also provides limited exploration of important contextual and cultural factors, such as stigma, family support, and beliefs surrounding disability, which may significantly influence the use and acceptance of HAs and APs. Furthermore, it lacks detail regarding the specific types and contextual appropriateness of HA and AP in the rural northern Thai setting. These limitations affect the external validity of the study and constrain the applicability of its findings across different disability types and geographic areas. Future research should conduct a longitudinal approach to examine the long-term impact of HA and AP use on quality of life, allowing for a deeper understanding of users’ evolving experiences over time.

### Conclusions

Enhancing access to HAs and APs for persons with disabilities in rural communities requires a comprehensive and collaborative approach. These supports are essential for improving quality of life by promoting safety, efficiency, independence, and participation in daily activities. Active engagement of families and communities is equally important, as they play a crucial role in mobilizing local resources to support budgeting, information sharing, accessibility, and ongoing maintenance. To ensure sustainable and equitable access, both local and central governments must work to address barriers, strengthen community support systems, and implement inclusive policies that facilitate the provision of HAs and APs. In addition, further studies are needed to explore the long-term impacts of these interventions, helping to develop contextually appropriate and effective solutions for persons with disabilities in underserved rural areas.

## Supplementary material

10.2196/79040 Multimedia Appendix 1 Daily living activities supported by home adaptations and assistive products (n=84).

10.2196/79040 Multimedia Appendix 2 Participants’ perspectives on barriers to modifying home, living environment, and using assistive products (n=84), including participants without experience home adaptations and assistive products (n=48) and participants with experience home adaptations and assistive products (n=36).

## Abbreviations

AP: assistive product
HA: home adaptation
NGO: nongovernmental organizations
WHO: World Health Organization
