# Supplementary figures and images for: Perspectives of Persons With Disabilities Toward Home Adaptations and Assistive Products in Rural Northern Thailand: Comparative Study
Source: Asian Pac Isl Nurs J. 2025 Oct 23;9:e79040. doi: 10.2196/79040 (PMC12548968; doi:10.2196/79040)

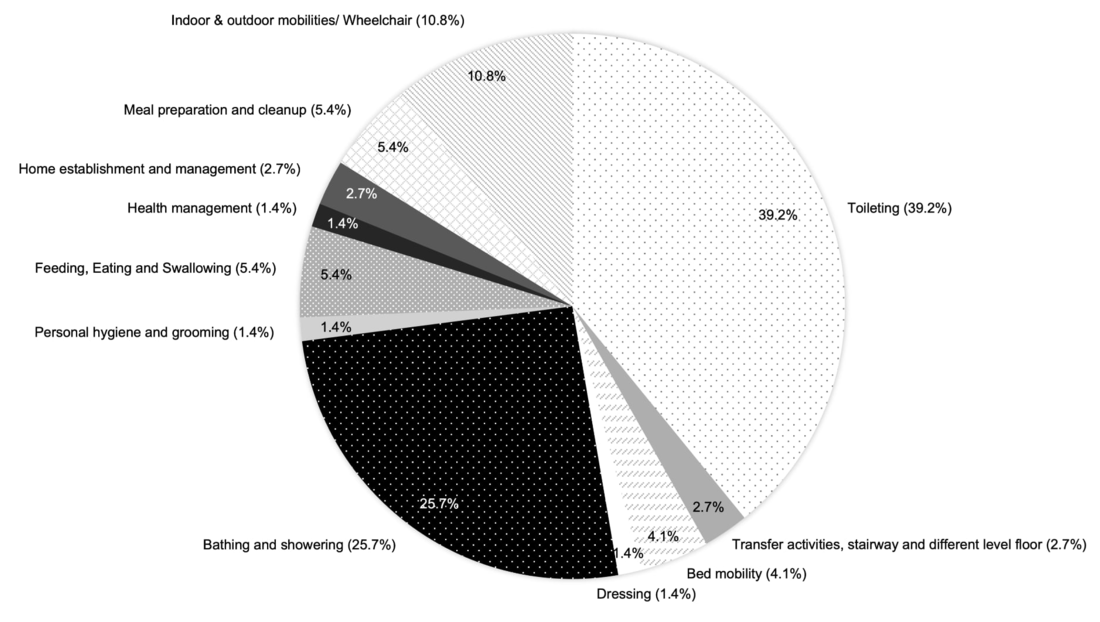

Supplement: Multimedia Appendix 1 [file apinj-v9-e79040-s001.png]
