# Supplementary material for: Perspectives of Persons With Disabilities Toward Home Adaptations and Assistive Products in Rural Northern Thailand: Comparative Study
Source: Asian Pac Isl Nurs J. 2025 Oct 23;9:e79040. doi: 10.2196/79040 (PMC12548968; doi:10.2196/79040)
Supplement: Multimedia Appendix 2 [file apinj-v9-e79040-s002.docx]

| Perspective and subgroup | | Strongly disagree, n (%) | Disagree, n (%) | Neutral, n (%) | Agree, n (%) | Strongly agree, n (%) |
| --- | --- | --- | --- | --- | --- | --- |
| Do you think that the cost of modifying your home or living environment, and using various APs^a^ is a significant barrier for you? | |  |  |  |  |  |
|  | Without experience HA^b^ and AP | 0 (0) | 1 (2) | 0 (0) | 11 (23) | 36 (75) |
|  | With experience HA and AP | 0 (0) | 0 (0) | 2 (6) | 8 (22) | 26 (72) |
| Do you think the lack of information about modifying your home, living environment, and use of APs is a barrier for you? | |  |  |  |  |  |
|  | Without experience HA and AP | 0 (0) | 3 (6) | 1 (2) | 39 (82) | 5 (10) |
|  | With experience HA and AP | 1 (3) | 0 (0) | 4 (11) | 26 (72) | 5 (14) |
| Do you think the difficulty in accessing services for assessing home modifications and using assistive devices is a barrier for you? | |  |  |  |  |  |
|  | Without experience HA and AP | 1 (2) | 2 (4) | 5 (10) | 34 (71) | 6 (13) |
|  | With experience HA and AP | 0 (0) | 1 (3) | 3 (8) | 28 (78) | 4 (11) |
| Do you think that maintaining a modified home and environment or using APs is a significant barrier for you? | |  |  |  |  |  |
|  | Without experience HA and AP | 0 (0) | 4 (8) | 2 (4) | 33 (68) | 10 (20) |
|  | With experience HA and AP | 0 (0) | 4 (11) | 3 (8) | 16 (45) | 13 (36) |

^a^AP: assistive product.

^b^HA: home adaptation.
